# Warming yang method in traditional Chinese medicine for depression: A protocol for systematic review and meta-analysis

**DOI:** 10.1097/MD.0000000000023919

**Published:** 2020-12-24

**Authors:** Jiashuai Deng, Changhong Wang, Yong Jiang

**Affiliations:** Chengdu University of Traditional Chinese Medicine, School of Basic Medical Sciences.

**Keywords:** depression, meta-analysis, protocol, traditional Chinese medicine, Warming yang method

## Abstract

**Background:**

Depression is a chronic psychological disease with low emotion, interest, cognition, thinking and disorder of physiological function, even numbness, or hallucination, delusion and other psychotic symptoms. Traditonal Chinese medicine plays an important role in the treatment of mental diseases in China and East Asia. The aim of this study is to assess the efficacy and safety of warming yang method in traditional Chinese medicine for depression.

**Methods:**

We search the following databases: PubMed, the Cochrane Library, Chinese Science and Technique Journals Database, Chinese Biomedical Literature Database, Excerpt Medica Database, MEDLINE, Chinese National Knowledge Infrastructure Database, and the Wanfang Database. Other sources will also be searched like Google Scholar and gray literatures. All databases mentioned above are searched from the start date to November 2020. Randomised controlled trials will be included which recruiting depression participants to assess the efficacy and safety of warming yang method in traditional Chinese medicines against controls (placebo or other therapeutic agents). Primary outcomes will include the reduction ratio of Hamilton Depression Rating Scale and security index. Secondary outcomes include the Yang deficiency improves. Two authors will independently scan the searched articles, extract the data from attached articles and import them into Endnote X8 and use Microsoft Excel 2013 to manage data and information. We will assess the risk of bias by Cochrane tool of risk of bias. Disagreements will be resolved by consensus or the participation of a third party. All analysis will be performed based on the Cochrane Handbook for Systematic Reviews of Interventions. The meta-analysis in this review will use RevMan 5.3 software.

**Results:**

The study is aim to evaluate the efficacy and safety of warming yang method in traditional Chinese medicine for depression.

**Conclusion:**

This study of the meta-analysis could provide evidence for clinicians and help patients to make a better choice.

**INPLASY registration number:**

INPLASY2020110129

## 1 Introduction

Depression is a chronic psychological disease with genetic tendency caused by a variety of factors, the main symptoms are low emotion, interest, cognition, thinking and disorder of physiological function, even numbness, or hallucination, delusion and other psychotic symptoms. Some patients had suicide, or accompanied by obvious anxiety and (or) provocation, and even aggressive behavior.^[1]^ About 13% to 20% of people have experienced depression in their lifetime, and the incidence rate in the whole life is 6.1% to 9.5%. The incidence all over the world is 12% to 17%.^[2]^ According to the WHO report, depression may become the second largest disease after heart disease in 2020.^[3]^ The pathogenesis of this disease is still unclear, the main point of view is the following aspects:1. 5-hydroxytryptamine (5-HT), also known as serotonin, is an important central neurotransmitter. Together with other central neurotransmitters, 5-HT participates in the nerve transmission of the central nervous system, and participates in behavioral activities, emotion, appetite regulation, etc. Studies have confirmed that the decline of 5-HT neurotransmitter not only leads to the formation of mood disorders, including depression and anxiety, but also induces depression by affecting the activities of other neurotransmitters.^[4]^ 2. Dopamine belongs to catecholamines. It is an important monoamine neurotransmitter. It can regulate physical, mental, endocrine and cardiovascular activities. Studies have shown that the lack of dopamine production in the body is also closely related to the incidence of depression.^[5]^ 3. Norepinephrine also belongs to catecholamines, which is not only a neurotransmitter, but also a hormone. Many studies have shown that NE concentration in hypothalamus is decreased in patients with depression, which indicates that depression is associated with central noradrenergic depression. 4. Functional changes of hypothalamus pituitary adrenal axis in patients with depression.^[6]^ 5. Clinical observation of some depression patients with obvious family history, the survey found that the prevalence of depression in these people is 10–30 times higher than the general population, the closer the blood relationship, the higher the probability of disease. 6. Clinical studies have found that the immune function of patients with depression has also changed. The levels of interleukin (IL)-1 other cytokines are significantly higher than those of healthy people. The expression of some pro-inflammatory factors such as IL-1B, IL-6, interferon-α and tumor necrosis factor-α in the central nervous system increased, while the anti-inflammatory factors IL-4, IL-8 and IL-10 increased after using anti-depressant drugs.^[7]^ In recent years, commonly clinical antidepressant chemicals include 5-HT reuptake blockers (such as fluoxetine, paroxetine and sertolin), and 5-th and norepinephrine (NE) dual inhibitors (such as venlafaxine, Flupentixol, melitracen tablets and mirtazapine). Although 5-HT and NE antidepressants have good antidepressant effect, there are still adverse reactions of different severity, and patients’ compliance is low.^[8]^ Traditional Chinese medicine is 1 of the most common and effective treatment that using the medicine extracted from botanical or mineral sources to cure diseases. In China and East Asia, traditional Chinese medicine has a long history in the treatment of various diseases and it has been involved in the intervention of depression for decades, which greatly enriches the treatment of depression and achieves better curative effect.^[9–14]^ Fortheremore, many single medicine and decoctions have been proved effectives on depression from pharmacological aspects, not only in clinical observation.^[15–20]^ Yang (or Yang qi) is a kind of special and significant concept in traditional Chinese medicine (TCM), it can warm body, produce a feeling of excitement, make qi and blood flow smoothly. So traditional Chinese Medicien holds that Yang deficiency is the cause of depression. Many TCM clinicians discuss the theory of warming yang method and use some single medicine and decoctions by the theory to treat depression and achieve good curative effect.^[21–27]^ Human beings are a complex creature, and depression has many pathogenic factors, so we must take a holistic approach to treat it. This is fully a close correspondence with the characteristics of TCM syndrome differentiation. However, the efficacy and safety of warming yang method in traditional Chinese medicine on depression still needs to be verified and no comprehensive assessments have been reported in recent years. Therefore, our aim is to evaluate the therapeutic effect of warming yang method in traditional Chinese medicine, and support clinicians to make better decisions.

## 2 Methods and analysis

### 2.1 Objectives and registration

This study will evaluate the efficacy and safety of warming yang method in traditional Chinese Medicine on depression. The protocol has been registered in International Platform of Registered Systematic Review and Meta-analysis Protocols (INPLASY) as INPLASY2020110129. And the article will adhere to the Preferred Reporting Items for Systematic Reviews and Meta-Analyses Statement (PRISMA-P reporting guidelines).^[28]^

### 2.2 Eligibility criteria

#### 2.2.1 Types of studies

Randomized controlled trials (RCTs) in Chinese and English will be enrolled in this system review. We will exclude non RCT, quasiRCT, reviews, experimental studies, case reports, cohort studies, expert experience, duplicate publication and the included study that the data is incomplete or missing.

#### 2.2.2 Types of participants

All patients diagnosed with depression will be included and there are no restrictions on their nationality, occupation, educational background, belief, age, body or race.

#### 2.2.3 Types of interventions

Warming yang method in traditional Chinese medicine will be included and there are no restrictions on single medicines, decoctions, dosage. The comparsions will be either with other therapeutic agents or placebo.

### 2.3 Types of outcome measures

#### 2.3.1 Primary outcomes

(1) The reduction ratio of Hamilton Depression Rating Scale (HAMD-17)^[29]^
(2) security index

#### 2.3.2 Secondary outcomes

(1) Yang deficiency improves

### 2.4 Search methods

#### 2.4.1 Electronic searches

We will retrieve the following databases: PubMed, the Cochrane Library, Chinese Science and Technique Journals Database, Chinese Biomedical Literature Database, Excerpt Medica Database, MEDLINE, Chinese National Knowledge Infrastructure Database, and the Wanfang Database. And we will search all the above databases from the available date of inception to November 2020.

#### 2.4.2 Other sources

The search the reference lists of reviews and retrieve articles will be searched for additional studies identify further studies, such as Google Scholar. We will also conduct a manual search of relevant conference reports and contact experts and corresponding authors in the field to obtain important information that cannot be obtained by the above retrieval.

#### 2.4.3 Search strategy

We will search for articles guided by Cochrane Handbook. The Search strategy for PubMed is shown in Table 1, and similar strategies will be built and applied for other electronic databases.

**Table 1:** The search strategy for Pubmed.

Number	Search terms
#1	Depression [MeSH Terms]
#2	traditional Chinese medicine [Title/Abstract]OR Chinese medicine [Title/Abstract]OR Chinese herbal medicine [Title/Abstract]
#3	RCT[Title/Abstract]OR randomized controlled trial [Title/Abstract]
#4	Efficacy [Title/Abstract] OR Safety [Title/Abstract]
#5	#1and #2 and #3 and #4

### 2.5 Data collection and analysis

#### 2.5.1 Selection of studies

**Figure 1:**
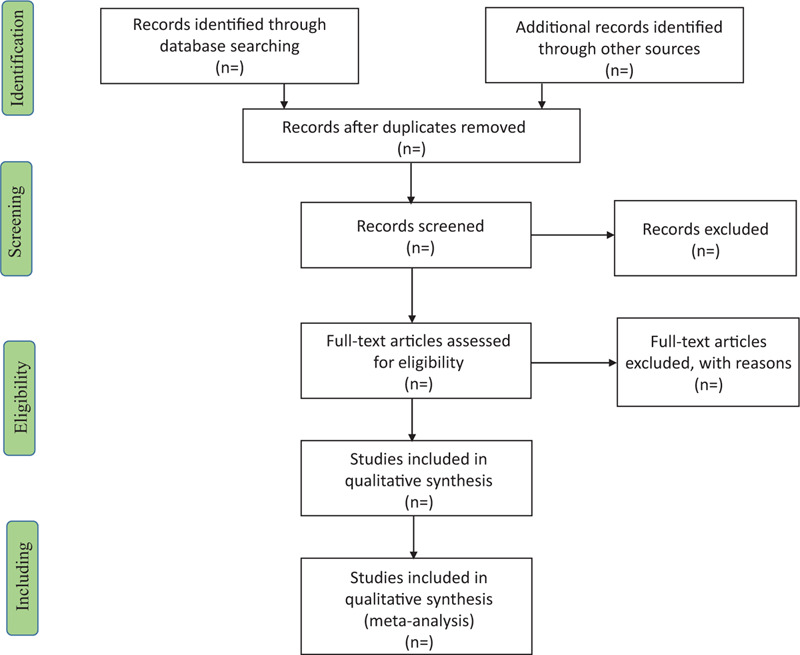
Flow chart of study selection.

The researchers will discuss and determine the screening criteria in groups before searching for studies. The data will be imported into Endnote X8 after obtaining relevant literatures from the above databases. Two reviewers (JD and CW) will independently screen all article's titles, abstracts and keywords. Then, the unqualified literature like duplicate documents will be excluded and the final included literature was determined by reading the full text. If there is any disagreement in the process, the third research member (YJ) will resolve the inconsistency and check the final included studies. The selection process will be shown in the Preferred Reporting Items for Systematic Review and Meta-analysis flow chart in Figure 1.

#### 2.5.2 Data extraction and management

Two authors (JD and CW) will extract relevant information independently from all studies that we obtained and import them into Microsoft Excel 2013 for data and information management. Data for collection include inclusion and exclusion, disease diagnosis, Hamilton rating, specific Chinese syndrome differentiation, relevant symptoms, treatment duration, study design, sample size, age, interventions and details about the control group, follow-up, outcomes, findings, and adverse event details. The disagreement will be settled by another reviewer (YJ). We will extract for informations as follows:

#### 2.5.3 Assessment of the risk of bias in the included studies

2 authors (JD and CW) will use the Cochrane tool of risk of bias to assess the risk of bias independently. The disagreement will be settled by another reviewer (YJ). We will access the following contents: selection bias (random sequence generation, and allocation concealment), performance bias (blinding of participants and personnel), detection bias (blinding of outcome assessment), attrition bias (incomplete outcome data), reporting bias (selective outcome reporting), and other bias (other sources of bias). Studies will be evaluated high, low and unknown.

#### 2.5.4 Measurement of the treatment effect

The meta-analysis in this review will use RevMan 5.3 software. Continuous variables will be reported as mean difference with 95% confidence intervals (CIs). For different measurement scales, we will use the standardized mean difference analysis with 95% CIs. Categorical variables will be summarized as risk ratios or odds ratio with 95% CIs. All analyses will be conducted in accordance with the Cochrane Handbook for Systematic Reviews of Interventions.^[30]^

#### 2.5.5 Dealing with missing data

For studies in which the data are missing or insufficient, we will try to obtain informations by contacting the corresponding author of the study. If contact is lost, we will build our analysis on the available data.

#### 2.5.6 Assessment of heterogeneity

Heterogeneity will be assessed by visual inspection of the forest and tested by standard Chi-squared statistic and a significance level of 0.1. Furthermore, the *I*^2^ statistic will be used to examine heterogeneity to quantify inconsistency. Fixed or random effects models will be performed in meta-analysis. If *I*^2^ > 0.5, the random effects models will be used.^[30]^

#### 2.5.7 Assessment of reporting biases

If more than ten studies that we include in the meta-analysis, funnel plots and Egger test will be used to assess the reporting bias, like publication bias. We will evaluate the potential for small study bias by using funnel plots. The asymmetry of funnel plots will show the possible small research effects.^[31,32]^

#### 2.5.8 Subgroup analysis

If heterogeneity is detected, subgroup analysis will be performed to explore the differences in the methodologic quality, age, race/ethnicity, and types of Chinese medicine.

#### 2.5.9 Sensitivity analysis

Sensitivity analysis will be performed to examine the robustness of the result if there are sufficient studies included. The factors on effect are as follows:

methodologic quality: analysis will be performed excluding studies of poor methodologic quality
sample size: analysis will be performed excluding small sample size studies
diagnostic criteria: analysis will be performed in studies of the same diagnostic criteria

#### 2.5.10 Confidence in cumulative evidence

The level of evidence of the results will be evaluated by a methodology based on the Grading of Recommendations Assessment, Development and Evaluation. And we will assess the evidence quality based on several factors including research limitations, effect consistency, imprecision, indirectness, and publication bias. The assessments will be categorized as high quality, medium quality, low quality and very low quality.

## 3 Discussion

With the increasing pressure of life, the incidence of depression is higher. However, because of the side effects of chemical medicines, high recurrence rate and narrow antidepressant spectrum, the compliance of patients is not high.^[33]^ Traditional Chinese medicine possesses the characteristics of multi target and systematization, small side effects and simple and easy operation, so TCM could help make up for the shortcoming of chemical medicines. In TCM, depression is known as “Bai he disease,” “Yu disease,” and the cause of the disease is yang deficiency. TCM using warming yang method to treat depression can comprehensively stimulate the body's qi and regulate the balance of qi and blood, yin and yang. Although there is no clear research on the mechanism of depression, it is urgent for doctors to relieve the pain of patients through effective treatment.

Therefore, we will assess the efficacy and safety of warming yang method in traditional Chinese medicine for depression by using systematic review and meta-analysis. The results of this study can provide a possible ranking for TCM treatment of depression. We hope that these results will provide clinicians with the evidence for TCM treatment of depression and help clinicians to make the best choices for patients.

## Author contributions

**Conceptualization:** Jiashuai Deng, Yong Jiang.

**Data curation**: Jiashuai Deng, Changhong Wang.

**Formal analysis**: Jiashuai Deng.

**Funding acquisition**: Yong Jiang.

**Methodology:** Jiashuai Deng, Changhong Wang.

**Project administration:** Jiashuai Deng, Changhong Wang.

**Supervision**: Yong Jiang.

**Writing – original draft:** Jiashuai Deng, Changhong Wang.

**Writing – review & editing:** Jiashuai Deng, Yong Jiang.
